# Neferine inhibits proliferation and collagen synthesis induced by high glucose in cardiac fibroblasts and reduces cardiac fibrosis in diabetic mice

**DOI:** 10.18632/oncotarget.11225

**Published:** 2016-08-11

**Authors:** Xue Liu, Xiuhui Song, Jianjun Lu, Xueying Chen, Ershun Liang, Xiaoqiong Liu, Mingxiang Zhang, Yun Zhang, Zhanhui Du, Yuxia Zhao

**Affiliations:** ^1^ The Key Laboratory of Cardiovascular Remodeling and Function Research, Chinese Ministry of Education and Chinese Ministry of Public Health, Qilu Hospital, Shandong University, Jinan, Shandong 250012, China; ^2^ Department of Traditional Chinese Medicine, Qilu Hospital, Shandong University, Jinan, Shandong 250012, China; ^3^ The People's Hospital of Jimo City, Qingdao, Shandong 266200, China; ^4^ The People's Hospital of Qihe City, Dezhou, Shandong 251100, China; ^5^ Department of Cardiology, Qilu Hospital, Shandong University, Jinan, Shandong 250012, China

**Keywords:** neferine, diabetes mellitus, cardiac fibrosis, TGF-β1

## Abstract

Cardiac fibrosis is a common pathological process accompanying diabetes mellitus. In this report, we studied the effects of neferine (a major bisbenzylisoquinline alkaloid derived from lotus embryos) on cardiac fibrosis induced by diabetes mellitus, as well as the underlying molecular pathways. *In vivo*, type 1 diabetes mellitus was induced in mice by administering streptozotocin. Diabetic mice were treated with neferine through oral gavage, and cardiac function was assessed using echocardiography. Total collagen deposition was assessed by Masson's trichrome and Picrosirius staining. *In vitro*, cardiac fibroblasts were cultured in normal or high-glucose medium with or without neferine. Neferine attenuated left ventricular dysfunction and remodeling and reduced collagen deposition in diabetic mice. *In vitro*, neferine inhibited cardiac fibroblast proliferation, migration, and differentiation into myofibroblasts. In addition, neferine reduced high-glucose-induced collagen production and inhibited TGF-β1-Smad, ERK and p38 MAPK signaling activation in cardiac fibroblasts. These results suggest that neferine may have antifibrogenic effects in diabetes-related cardiac fibrosis.

## INTRODUCTION

Diabetes mellitus (DM) is a global health concern [1]. The burden of diabetes as a major cause of premature illness and death is mostly due to the associated increased risk of cardiovascular disease, cardiac remodeling and heart failure. Cardiac fibrosis is reported to be a key pathogenic component of cardiovascular diseases [2]. Specifically, cardiac fibrosis contributes to cardiac remodeling, increases myocardial stiffness, reduces the pumping capacity of the heart, and eventually leads to heart failure [3]. Nevertheless, no curative treatment for cardiac fibrosis has been developed so far.

Cardiac fibroblasts (CFs) are the predominant cell type in the heart, and are responsible for the basal deposition and degradation of the extracellular matrix (ECM) in the normal heart [4]. As the main matrix-producing cells, CFs are critically involved in all cardiac fibrotic conditions. In response to various stimuli, CFs may proliferate, migrate, differentiate into myofibroblasts, generate or degrade the ECM, secrete cytokines and growth factors, and so on. High glucose (HG) in the blood (hyperglycemia), the main feature of diabetes mellitus, can stimulate collagen deposition by inducing CF proliferation and activation *in vitro* [5, 6]. On the basis of these concepts, inhibiting the activation of CFs could be a viable strategy for treating cardiac fibrosis.

“Lianzixin,” the seed embryo of *Nelumbo nucifera* (Gaertn.), has been commonly used in traditional Chinese medicine as a sedative, antipyretic and hemostatic agent. Neferine is a major bisbenzylisoquinline alkaloid derived from this plant, along with liensinine and isoliensinine [7]. Neferine has been reported to have a variety of biological and pharmacological effects, such as anti-hypertensive, anti-arrhythmic [8], anti-agglutinating [9], anti-thrombotic [9], antioxidant, anti-inflammatory [10], neuroprotective [11], anticancer [12–14], negative inotropic and vascular-smooth-muscle-relaxing effects [15]. In addition, neferine exerts antifibrotic effects. Zhao et al. found that neferine attenuated bleomycin-induced pulmonary fibrosis *in vitro* and *in vivo* [7]. Niu et al. demonstrated that neferine significantly inhibited amiodarone-induced pulmonary fibrosis [16]. A recent study revealed that neferine had an antifibrotic effect on CCl_4_-induced hepatic fibrosis in mice, which may have been partly due to the reduced expression of transforming growth factor-β1 (TGF-β1) in the liver [17]. Hence, the current study was designed to determine whether and by what molecular pathways neferine could attenuate cardiac fibrosis induced by HG in CFs, and whether neferine could thus serve as an alternative and safe drug for clinical applications.

## RESULTS

### Neferine inhibited HG-induced proliferation of CFs

**Figure 1:**
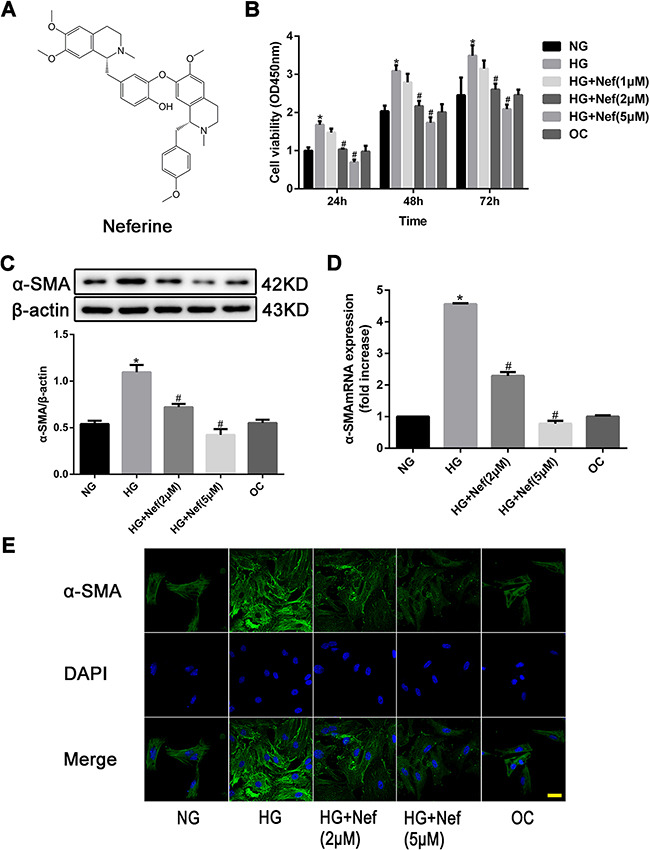
Neferine inhibited high glucose (HG) induced proliferation of cardiac fibroblasts (CFs) **A.** Chemical structure of neferine. **B.** CFs were treated with normal glucose medium (NG), HG medium, HG medium with various concentrations of neferine, and normal glucose medium with mannose (OC) for 24, 48 and 72 h. Cell viability was measured by CCK-8 assay. **C.** Western blot analysis of α-SMA protein expression. **D.** PCR analysis of *α-SMA* mRNA expression. **E.** Immunocytofluorescence results of α-SMA. Scale bar: 25 μm. α-SMA was stained green; nuclei stained with DAPI were blue. NG: 5.6 mM glucose, HG: 30 mM glucose, HG+Nef (2μM): 30 mM glucose + 2 μM neferine, HG+Nef (5μM): 30 mM glucose + 5 μM neferine, OC: 5.6 mM glucose + 27.5 mM mannose. Data were mean ± SD of three independent experiments. *P<0.05 compared with the NG group; #*P*<0.05 compared with the HG group.

The structure of neferine is depicted in Figure 1A. CFs were cultured in HG medium with varying concentrations of neferine (1, 2, or 5 μM). As shown in Figure 1B, CCK-8 assays were carried out at different time points (24, 48, and 72 h). Compared with normal glucose (NG) and osmotic control (OC) treatments, HG (30 mM) treatment significantly increased the proliferation of CFs in a time-dependent manner (*P*<0.05). HG-induced CF proliferation was markedly attenuated by neferine treatment at either 2 or 5 μM compared with vehicle treatment. However, 1 μM neferine did not inhibit HG-induced proliferation of CFs. Therefore, 2 and 5 μM neferine were used in the remaining experiments.

Western blot analysis revealed that the protein expression of alpha-smooth muscle actin (α-SMA) was significantly greater in CFs exposed to HG than in those exposed to NG or OC, (both *P*<0.05, Figure 1C). The expression of α-SMA under HG conditions was lower in CFs treated with neferine at 2 or 5 μM than in those treated with HG only (both *P*<0.05). The mRNA expression of *α-Sma* was evaluated quantitatively by RT-PCR, and the results were consistent with those of the Western blot analysis (Figure 1D). Immunofluorescence staining confirmed that the protein expression of α-SMA in CFs exposed to HG was attenuated by neferine treatment at 2 or 5 μM (both *P*<0.05, Figure 1E).

### Effect of neferine on cell cycle distribution in CFs

**Figure 2:**
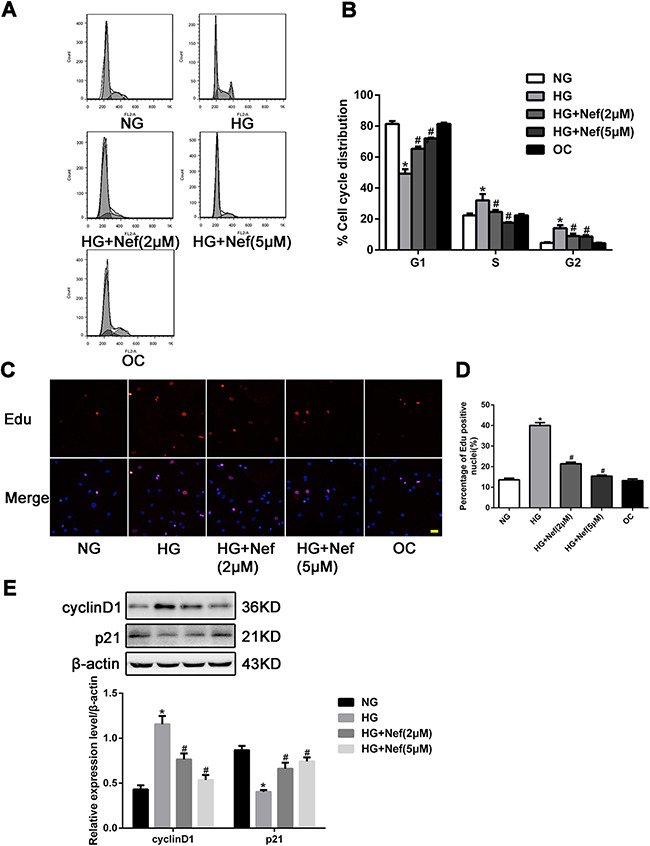
Effect of neferine on cell cycle distribution in CFs **A.** Neferine induced G1 cell cycle arrest in CFs. The distribution of cell cycle was assessed by flow cytometry. **B.** The percentage of CFs in each phase is shown as the mean ± SD. **C.** Laser confocal microscopy of Edu staining. Nuclei were stained blue with DAPI. The red indicated the cells undergoing proliferation. Scale bar: 25 μm. **D.** Edu positive index was expressed as a percentage of cell counts. **E.** Representative Western blot and quantitative analysis of cyclin D1 and p21 protein expression in CFs. NG: 5.6 mM glucose, HG: 30 mM glucose, HG+Nef (2μM): 30 mM glucose + 2 μM neferine, HG+Nef (5μM): 30 mM glucose + 5 μM neferine, OC: 5.6 mM glucose + 27.5 mM mannose. Data were mean ± SD of three independent experiments. *P<0.05 compared with the NG group; #*P*<0.05 compared with the HG group.

As shown in Figure 2A and 2B, HG induced a greater extent of CF proliferation than NG or OC (both *P*<0.05), by promoting more cells from G1 to S phase. Under HG conditions, neferine treatment (2 or 5 μM) for 48 h increased the proportion of cells in G1 phase and reduced the proportions in G2 and S phase. The percentages of CFs in G1 phase were 49.33%, 65.33% and 72.00% in the HG, HG+2μM neferine and HG+5μM neferine groups, respectively. Meanwhile, the percentages of cells in G2 phase were reduced to 9.00% (HG+2μM neferine) and 8.67% (HG+5μM neferine), from 14.00% in the HG group. The percentages of cells in S phase were 24.67% in the HG+2μM neferine group, 17.67% in the HG+5μM neferine group, and 32.00% in the HG group. The EdU incorporation assay confirmed that the percentages of CFs in S phase were lower in the HG+2μM neferine and HG+5μM neferine groups than in the HG group (*P*<0.05, Figure 2C and 2D).

To examine the underlying molecular pathways responsible for neferine-induced G1 arrest, we next analyzed cyclin D1 and p21 protein levels by Western blot analysis. The protein expression of cyclin D1 was downregulated in the HG+neferine groups (both 2 and 5 μM) compared with the HG group, while the protein level of p21 was upregulated following neferine treatment (Figure 2E).

### Neferine reduced the collagen deposition, downregulated the protein expression of TGF-β1, and inhibited the migration of CFs

**Figure 3:**
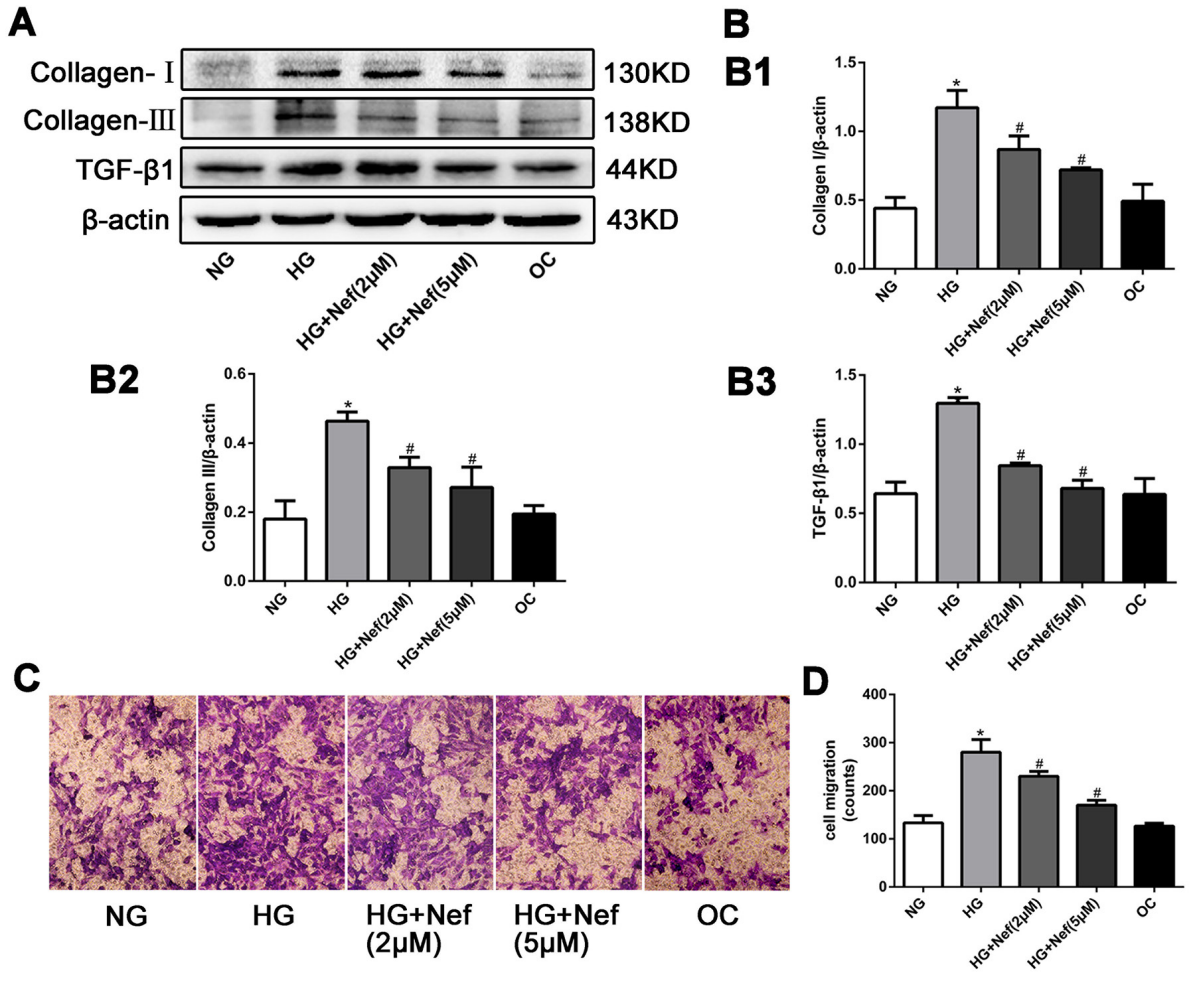
Neferine reduced the collagen deposition, down-regulated the protein expression of transforming growth factor β1 (TGF-β1), and inhibited the migration of CFs **A.** Western blot analysis of collagen I and III and TGF-β1 protein levels. **B.** Quantitative analysis of the protein expression of collagen I and III and TGF-β1. **C.** Transwell migration assay showed that neferine attenuated HG induced CFs migration. CFs were cultured in HG medium with neferine in 8-μm-pore-sized Transwell chamber for 10 h. CFs on the external surface of Transwell chamber were dyed with crystal violet and photographed under a microscope. **D.** Quantification analysis of migration CF numbers in per filed of Transwell. NG: 5.6 mM glucose, HG: 30 mM glucose, HG+Nef (2μM): 30 mM glucose + 2 μM neferine, HG+Nef (5μM): 30 mM glucose + 5 μM neferine, OC: 5.6 mM glucose + 27.5 mM mannose. Data were means ± SD of three independent experiments. *P<0.05 compared with the NG group; #*P*<0.05 compared with the HG group.

The protein expression of collagen I and III and TGF-β1 in CFs were greater in the HG group than in the NG and OC groups. Neferine treatment at 2 and 5 μM attenuated the increases of collagen I, III and TGF-β1 expression induced by HG (Figures 3A and 3B).

A migration assay demonstrated that HG promoted CF migration relative to NG and OC, while 2 and 5 μM neferine suppressed this increase (Figures 3C and 3D).

### Neferine attenuated diabetes-induced myocardial remodeling *in vivo*

**Figure 4:**
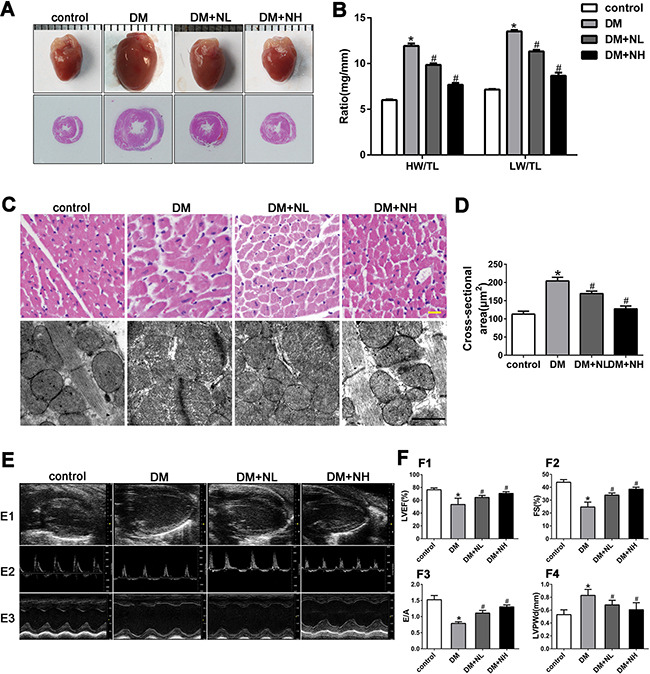
Neferine attenuated diabetes-induced myocardial remodeling *in vivo*. **A.** Representative photographs of mice heart and HE staining of the hearts **B.** Heart weight/tibial length (HW/TL) and lung weight/tibial length (LW/TL) ratios. **C.** Representative hematoxylin-eosin (HE) staining of left ventricular (LV) transverse sections. Scale bar: 20 μm. Transmission electron micrographs of cardiomyocytes. Scale bar: 2 μm. **D.** Quantification of cross-sectional area of cardiomyocytes from HE stained sections. **E. E1**: Representative 2D echocardiograms. **E2**: Representative pulsed-wave Doppler echocardiograms of mitral inflow. **E3**: Representative M-mode echocardiograms. **F. F1**: LV ejection fraction (LVEF). **F2**: Fractional shortening (FS). **F3**: The ratio of peak early to late diastolic filling velocity (E/A ratio). **F4**: LV posterior wall thickness at diastole (LVPWd). control: normal mice; DM: diabetic mellitus; DM+NL: DM mice with neferine administered at a dose of 60 mg/kg/day by gavage. DM+NH: DM mice with neferine administered at a dose of 120 mg/kg/day by gavage. Data were mean ± SD of three independent experiments. *P<0.05 compared with the control group; #*P*<0.05 compared with the DM group.

We next performed an animal experiment to determine the effects of a low (60 mg/kg/day, ‘NL’) or high dose of neferine (120 mg/kg/day, ‘NH’) on streptozotocin-induced diabetic mice. Mice were divided into four groups: control, DM, DM+NL and DM+NH. Compared with citrate (control), streptozotocin induced rapid hyperglycemia in mice, beginning one week after injection. Blood glucose, blood pressure, and heart rate were measured, as shown in Table 1. Heart size, the ratio of heart weight to tibia length (HW/TL), and the ratio of lung weight to tibia length (LW/TL) were comparable among the four groups (Figures 4A and 4B). HW/TL and LW/TL were significantly higher in the DM group than in the control group. However, both low- and high-dose neferine treatments reduced the hyperglycemia-induced increases in HW/TL and LW/TL. DM mice had greater cross section area of myocytes in the left ventricle than control mice (*P*<0.05), but this effect was attenuated by both low- and high-dose neferine treatments (Figure 4C and 4D).

**Table 1:** Blood glucose, blood pressure, and heart rate measurement

	Blood glucose(mM)	SBP(mmHg)	MBP(mmHg)	DBP(mmHg)	HR(pm)
**control**	9.99±0.75	96±2	89±2	70±2	595±5
**DM**	27.98±1.00*	119±4*	109±4*	86±4 *	509±10*
**DM+ NL**	24.98±1.48	110±6	100±3	78±4	518±5
**DM+ NH**	24.25±0.93	105±5	99±3	75±2	529±8

SBP, systolic blood pressure; MBP, blood pressure; DBP, diastolic blood pressure; and HR, heart rate. N=8 mice for each group. control: normal mice. DM: diabetic mellitus. DM+NL: DM mice received neferine at a dose of 60 mg/kg/day by gavage. DM+NH: DM mice received neferine at a dose of 120 mg/kg/day by gavage. All results were presented as mean±SD.

* *P*<0.05 versus the control group.

To assess the effects of neferine on myocardial ultrastructure, we used transmission electron microscopy to observe mitochondrial morphology and Z-line structures. Compared with control mice, mice in the DM group exhibited mitochondrial morphological alteration, crista fragmentation, and disorganized Z-line structures (Figure 4C). Neferine treatment at low or high doses attenuated the mitochondrial swelling, crista fragmentation and Z-line structural damage (Figure 4C).

Echocardiography was carried out to assess cardiac function (Figure 4E and 4F). The left ventricular ejection fraction (LVEF), fractional shortening (FS), and early-to-late mitral inflow velocity (E/A) ratio were all lower, while the left ventricular posterior wall at diastole (LVPWd) was greater, in diabetic mice than in control mice. In diabetic mice, neferine treatments at both low- and high-dose improved the cardiac function features (LVEF, FS, and E/A ratios). The LVPWd was lower in the DM+NL and DM+NH groups than in the DM group (*P*<0.05).

### Neferine prevented diabetes-induced cardiac fibrosis *in vivo*

**Figure 5:**
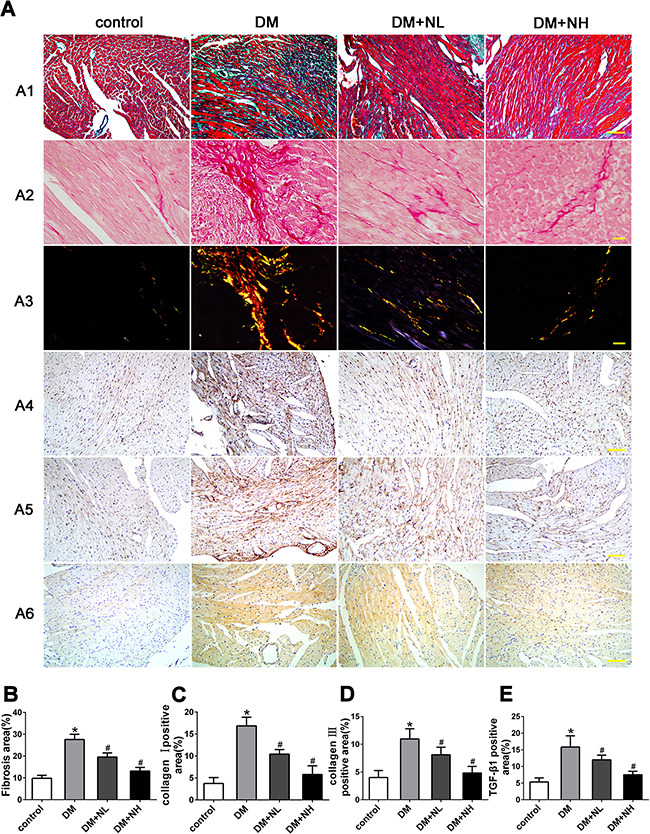
Neferine prevented diabetes-induced cardiac fibrosis in vivo **A. A1**: Representative Masson's trichrome staining. Scale bar: 25 μm. **A2-A3**: Picrosirius red staining of the myocardia. Scale bar: 10 μm. Immunostaining of collagen I **A4** and III **A5**. **A6**: Immunostaining of TGF-β1. Scale bar: 25 μm. **B.** Quantitative analysis of myocardial fibrosis. **C-E.** Quantitative analysis of protein expression of collagen I (C), III (D) and TGF-β1 (E). Control: normal mice. DM: diabetic mellitus. DM+NL: DM mice with neferine administered at a dose of 60 mg/kg/day by gavage. DM+NH: DM mice with neferine administered at a dose of 120 mg/kg/day by gavage. Data were mean ± SD of three independent experiments. *P<0.05 compared with the control group; #*P*<0.05 compared with the DM group.

Masson's trichrome and Picrosirius red staining of heart sections revealed that the ECM in the interstitial region of the myocardium was greater in diabetic mice than in control mice (Figure 5A1, A2, and A3). Quantitative analysis of Masson's trichrome staining demonstrated that collagen deposition was greater in diabetic mice than in control mice. Collagen deposition in the DM+NL and DM+NH groups was lower than in the DM group (Figure 5B). Figure 5A4, A5 and A6 depict the protein expression of collagen I and III and TGF-β1, respectively, as detected by immunohistochemistry in the four groups. Quantitative analysis revealed that collagen I, III and TGF-β1 expression were higher in diabetic mice than in controls. Neferine treatment at both low- and high-dose reduced the increment of collagen I, III and TGF-β1 protein expression induced by hyperglycemia (Figure 5C, 5D, and 5E).

### Signal transduction mechanism in CFs treated with neferine

**Figure 6:**
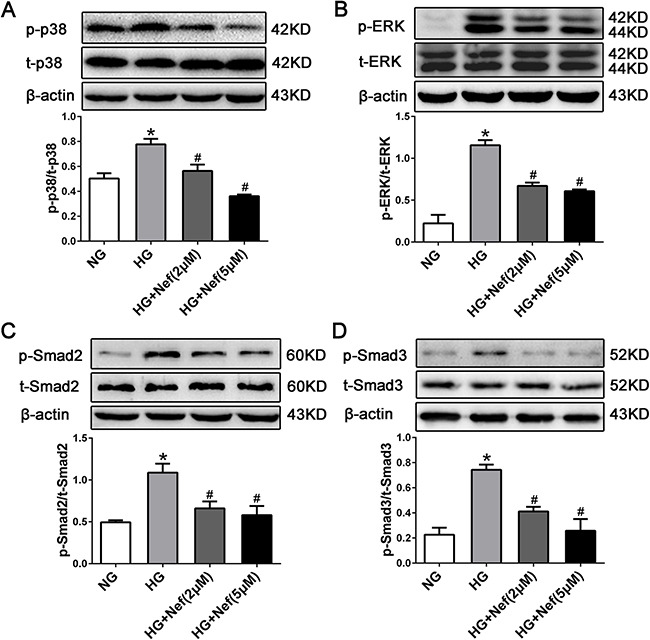
Neferine inhibited HG induced TGF-β1-Smads and the ERK and p38 MAPK signaling activation in CFs Western blot analysis of p-p38/t-p38 **(A)**, p-ERK/t-ERK (**B**), p-Smad2/t-Smad2 (**C**), and p-Smad3/t-Smad3 (**D**) protein expression in CFs. NG: 5.6 mM glucose, HG: 30 mM glucose, HG+Nef (2μM): 30 mM glucose + 2 μM neferine, HG+Nef (5μM): 30 mM glucose + 5 μM neferine. Data were mean ± SD of three independent experiments. *P<0.05 compared with the NG group; #*P*<0.05 compared with the HG group.

The potential signal transduction pathway involved in HG-induced cardiac fibrosis was detected *in vitro* by Western blot analysis. Higher levels of phospho(p)-p38, p-extracellular-regulated protein kinase (p-ERK), and p-Smad2/3 phosphorylation were detected in HG-treated CFs than in normal CFs (*P*<0.05). However, the HG-induced phosphorylation upregulations of p-p38, p-ERK, and p-Smad2/3 were attenuated by neferine treatment at 2 and 5 μM (Figure 6).

### Safety with neferine treatment

To ascertain whether neferine may induce adverse effect, we examined the liver and renal histology after neferine treatment in mice. As shown in Supplement Figure 1F, no histological abnormalities were found in these organs after neferine treatment, indicating that neferine is a safe compound to use in our animals.

## DISCUSSION

Cardiac fibrosis is one of the major pathological processes in diabetes, but specific pharmaceuticals directly targeting fibrosis are still lacking [18]. It has been documented that neferine prevents pulmonary and hepatic fibrosis [7, 16, 17]. Thus, in the present study, we evaluated whether neferine would prevent diabetes-associated cardiac fibrosis.

Cardiac fibrosis is characterized by the proliferation of CFs [19, 20]. In our *in vitro* studies, when CFs were cultured with HG (30 mM), CF proliferation increased, while neferine inhibited HG-induced CF proliferation, migration and differentiation into myofibroblasts. As the inhibition of cell proliferation is usually caused by cell cycle arrest, we examined the distribution of cells in different phases of the cell cycle by flow cytometry. Neferine caused G1 cell cycle arrest in CFs, as the percentage of CFs in G1 phase increased at the expense of cells in S phase following neferine treatment. Cyclin D1 and p21 are the proteins that regulate G1/S progression. Cyclin D1 promotes the G0/G1-S transition of the cell cycle, while p21 can prevent the movement of cells into and from the S subphase, causing G1 subphase arrest [21, 22]. We found that cyclin D1 protein expression decreased and p21 expression increased in CFs exposed to neferine. Thus, neferine might up/downregulate these cell cycle proteins to inhibit the proliferation of CFs.

Fibroblast-myofibroblast transdifferentiation increases in failing hearts [23]. Myofibroblasts that express α-SMA may be responsible for the deposition of the ECM, which replaces the normal tissue structure during fibrosis [24]. HG treatment was reported to promote the spontaneous differentiation of cardiac fibroblasts into myofibroblasts with increasing passage compared to low-glucose treatment [25]. In the present study, we examined the protein expression of α-SMA in CFs under HG conditions. The protein level of α-SMA increased in CFs cultured in HG, while treatment with neferine reduced this increase. This result suggested that neferine might attenuate fibroblast-myofibroblast transdifferentiation.

The most important pathological feature of cardiac fibrosis is the excess production of the ECM, mainly collagen types I and III, which can alter the structure and function of the heart [19]. Both *in vitro and in vivo*, we observed that HG accelerated the synthesis and deposition of collagen types I and III within the interstices of the myocardium, consistent with the results of a previous study [6], while neferine reduced collagen deposition. It has been reported that the proliferation and differentiation of CFs to the myofibroblast phenotype results in excessive secretion of ECM proteins [26]. Thus, the inhibition of CF proliferation and fibroblast-myofibroblast transdifferentiation by neferine, as mentioned above, might reduce the production of the ECM.

In the present study, we found that TGF-β1 expression was elevated in HG-treated CFs, and that neferine partially inhibited this increase. TGF-β1 is the most powerful cytokine that has been recognized to induce fibrosis. It performs many cellular functions, including promoting fibroblast proliferation, differentiation, migration, and ECM production [6]. Smads are intracellular signal transduction proteins of the TGF-β1 pathway and involved in pathological changes. In our *in vitro* experiment, hyperglycemia increased the phosphorylation of Smad2/3, while neferine reversed this effect. Thus, neferine may prevent myocardial fibrosis by downregulating the expression of the TGF-β1 and preventing TGF-β1-Smad pathway activation.

In addition to the TGF-β1-Smad pathway, MAPK signaling has also been identified as an important contributor to the fibrotic process [27]. The major MAPK signaling cascades, including ERK1/2, JNK and p38 MAPK, are activated in the regulation of collagen production by cardiac fibroblasts [6, 28]. In our study, Western blot analysis revealed that the phosphorylation of ERK and p38 was greater in CFs treated with HG than in those treated with NG, while these increments were reduced by both low- and high-dose neferine treatments. It has been documented that HG induces the synthesis of collagen in CFs by activating the ERK1/2 cascade, and that inhibition of ERK1/2 phosphorylation significantly reduces the mRNA and protein levels of collagen I and III [6]. This suggested that neferine could inhibit the diabetes-associated fibrotic response through the MAPK signaling pathway. In our study, the protein levels of matrix metalloproteinases and p-JNK were greater in CFs treated with HG than in those treated with NG, but neferine had no effect on the increments (Supplementary Figure S1C, S1D, and S1E).

In summary, our study illustrated that neferine could attenuate HG-induced cardiac fibrosis *in vitro and in vivo*. Neferine inhibited the proliferation and collagen synthesis of cardiac fibroblasts and improved myocardial function in diabetic mice. It seems that neferine exerted antifibrotic effects partly by inhibiting TGF-β1-Smad and the ERK and p38 MAPK signaling pathways. These findings suggested that neferine might have therapeutic potential as a treatment for cardiac fibrosis. Further studies of its effects *in vivo* will provide more information about its usefulness in treating diabetic cardiovascular disease.

## MATERIALS AND METHODS

This investigation conformed to the Guide for the Care and Use of Laboratory Animals published by the National Institutes of Health (DRR/National Institutes of Health, 1996), and all experimental protocols were approved by the relevant Ethics Committee of Shandong University for Animal Care and Use.

### Cell culture and treatments

CFs were isolated from neonatal mouse ventricular tissues by a method described previously [29]. CFs were identified by immunocytochemistry as Vimentin (+)/vWF (−) cells (Supplementary Figure S1B).

Neferine (Sigma) was dissolved in sterile cell culture-grade dimethylsulfoxide (DMSO). Working dilutions of neferine were made in culture medium immediately before use with a uniform 0.1% concentration of DMSO.

After starvation in serum-free medium for 24 h, CFs were incubated in DMEM containing 5.6 mM glucose (normal glucose; NG), 30 mM D-glucose (HG), 30 mM D-glucose plus 1 μM neferine, 30 mM D-glucose plus 2 μM neferine, 30 mM D-glucose plus 5 μM neferine, and 5.6 mM glucose plus 27.5 mM mannose (osmotic control; OC). Cells were harvested at 24 h, 48 h, and 72h.

### Immunocytochemistry

Following the different treatments, CFs were seeded onto pre-coated glass cover slips. The cells were fixed with 4% paraformaldehyde, permeabilized in 0.03% Triton X-100, blocked with 10% goat serum, and then incubated with a primary anti-α-SMA monoclonal antibody (Sigma, St. Louis, MO, USA) followed by secondary antibody (FITC-conjugated goat anti-mouse IgG, Jackson Laboratories). Nuclei were visualized with 4-6-diamidino-2-phenyl indole (DAPI, 5 mg/mL, Beyotime, Haimen, China). The results were visualized with a fluorescence microscope.

### Real-Time PCR

Total RNA was extracted from cultured cells and reverse-transcribed into cDNA by means of the Prime Script RT reagent kit (Takara, Dalian, China). Quantitative RT-PCR was conducted on an iQ5 Multi-color Real-Time PCR Detection System (Bio-Rad, Hercules, CA, USA) with SYBR Green Real-time PCR Master Mix (Takara, Dalian, China). The PCR primer sequences were as follows.

*α-SMA*, forward, 5′-CTTCCAGCCATCTTTC ATTGG-3′, reverse, 5′-ATATCACACTTCATGATGCTGTTATAGGT-3′;

*β-actin*, forward, 5′-CACTGTGCCCATCTA CGA-3′, reverse, 5′-GTAGTCTGTCAGGTCCCG -3′

The relative changes in gene expression were calculated by the 2^−ΔΔCT^ method.

### Cell proliferation and cell cycle assay

Cell proliferation was measured with the Cell Counting Kit-8 (CCK-8, Beyotime, Haimen, China) and the Cell-Light^TM^ EdU assay (RiboBio, Guangzhou, China) according to the manufacturers' directions.

Cell cycle distribution was analyzed through the measurement of DNA content using flow cytometry. CFs were seeded into six-well plates (2×10^5^cells per well) for 24 h, treated with various concentrations of neferine for 48 h, fixed with precooled 70% ethanol at 4°C overnight, and then stained with propidium iodide (Multi Sciences Biotech Co., Ltd.) for 30 minutes in the dark. Acquisition and analysis were performed on a FACSCalibur system (Becton Dickinson, CA, USA), and BD FACS Diva software was used for data analysis.

### Western blot analysis

Total cell proteins were extracted in RIPA Lysis Buffer containing protein inhibitors (Beyotime, Guangzhou, China) according to the manufacturer's instructions. Membranes were incubated with primary antibodies at 4°C overnight and hybridized with horseradish peroxidase-conjugated goat anti-mouse or -rabbit IgG antibodies. The primary antibodies used were as follows: α-SMA, MMP2, MMP9, collagen I and III, and TGF-β1 (all Abcam, Cambridge, MA); JNK, p-JNK, p38 MAPK, p-p38 MAPK, ERK1/2, and p-ERK1/2 (all Cell Signaling Technology).

### Cell migration assay

Cell migration was examined in Transwell chambers. Briefly, 24-well Transwell chambers (Corning) equipped with 8-μm-pore-sized polycarbonate membranes were used. Serum-starved CFs (1×10^5^) were added to the upper chamber, while DMEM containing 10% serum, HG, and different concentrations of neferine was added to the lower chamber. The Transwell chamber was incubated for 10 h at 37°C in a humidified incubator with 5% CO2. The cells on the upper surface were removed with a cotton swab, fixed with 4% formaldehyde and stained with 0.1% crystal violet. Five randomly selected fields were photographed, and the migrated cells were counted.

### Animal experiments

All animal experimental protocols were approved by the Institutional Ethics Committee of Shandong University in accordance with the Guide for the Care and Use of Laboratory Animal published by the US National Institutes of Health and Shandong University. Eight-week-old C57BL/6J male mice were purchased from the Vital River Laboratory Animal Technology Co. Ltd (Beijing, China). Diabetes was induced by intraperitoneal injection of streptozotocin (Sigma, St Louis, MO, USA) dissolved in citrate buffer (pH 4.5) at 60 mg/kg body weight for five consecutive days. Control mice were injected with citrate buffer only. Whole blood glucose in mouse tail blood was detected with an Accu-Check Active glucometer (Roche). Mice with blood glucose concentrations higher than 18 mM were considered as diabetic animals and used in this study. The animals were randomly divided into four groups of eight animals each. Diabetic mice were divided into three groups: group 1, the diabetic control group (DM); group 2, which received neferine at a dose of 60 mg/kg/day (DM-NL); and group 3, which received neferine at a dose of 120 mg/kg/day (DM-NH). Neferine was administered twice per day by intragastric gavage for 12 weeks. Equivalent volumes of normal sodium were administered to the normal and DM control groups by gavage. Mice were anaesthetized and sacrificed at the end of the 12-week treatment.

### Non-invasive analysis of cardiac function

Left ventricular dimensions and cardiac function were assessed by echocardiography (Vevo770 imaging system) before mice were killed. Mice were anesthetized with isoflurane, and M-mode images at the level of the papillary muscles were obtained to measure the LVPWd, LVEF and FS. Pulsed-wave Doppler echocardiography was recorded to measure the E/A ratio.

### Transmission electron microscopy

Freshly dissected heart tissues were cut into 1-mm cubes and immersion-fixed with 2.5% glutaraldehyde overnight at 4°C, post-fixed with 1% buffered osmium tetroxide, and dehydrated in a series of graded ethanol concentrations. Specimens were double-immersed in uranyl acetate. The tissues were sectioned into 90-nm-thick slices with an LKB-8800 ultramicrotome (LKB-Produkter AB, Bromma, Sweden) and were examined by electron microscopy (model JEM-1200EX, Jeol Jem, Tokyo).

### Histology and immunohistochemistry

Myocardium samples were fixed with 4% paraformaldehyde for 24 h and then embedded in paraffin. Serial sections of 5 μm were cut and placed on polylysine-coated glass slides. Masson's trichrome, Sirius red, and hematoxylin-eosin staining were performed according to the manufacturers' instructions. Immunohistochemical staining for collagen I and III, and TGF-β1 were carried out with the SABC kit (Zhongshan, Beijing, China). The tissue sections were incubated with primary antibodies overnight at 4°C. Secondary antibody application and color development were performed according to the manufacturers' protocols. Data were analyzed in ImageProPlus6.0 (Media Cybernetics).

### Statistical analysis

The results shown are from at least three independent experiments. Data are expressed as the mean ± SD. The statistical significance was determined by one-way analysis of variance, followed by Tukey's post hoc test (SPSS statistical software package, version 16.0, SPSS, Chicago, IL, USA). A *P* value less than 0.05 was considered statistically significant.

## SUPPLEMENTARY FIGURE


