# Correction: Neferine inhibits proliferation and collagen synthesis induced by high glucose in cardiac fibroblasts and reduces cardiac fibrosis in diabetic mice

**DOI:** 10.18632/oncotarget.28214

**Published:** 2022-06-15

**Authors:** Xue Liu, Xiuhui Song, Jianjun Lu, Xueying Chen, Ershun Liang, Xiaoqiong Liu, Mingxiang Zhang, Yun Zhang, Zhanhui Du, Yuxia Zhao

**Affiliations:** ^1^The Key Laboratory of Cardiovascular Remodeling and Function Research, Chinese Ministry of Education and Chinese Ministry of Public Health, Qilu Hospital , Shandong University, Jinan, Shandong 250012, China; ^2^Department of Traditional Chinese Medicine, Qilu Hospital, Shandong University, Jinan, Shandong 250012, China; ^3^The People’s Hospital of Jimo City, Qingdao, Shandong 266200, China; ^4^The People’s Hospital of Qihe City, Dezhou, Shandong 251100, China; ^5^Department of Cardiology, Qilu Hospital, Shandong University, Jinan, Shandong 250012, China

**This article has been corrected:** In Figure 3C, cell migration images in the NG and OC groups were accidentally overlapped. The corrected Figure 3, produced using the original data, is shown below. The authors declare that these corrections do not change the results or conclusions of this paper.

Original article: Oncotarget. 2016; 7:61703–61715. 61703-61715. https://doi.org/10.18632/oncotarget.11225

**Figure 3:**
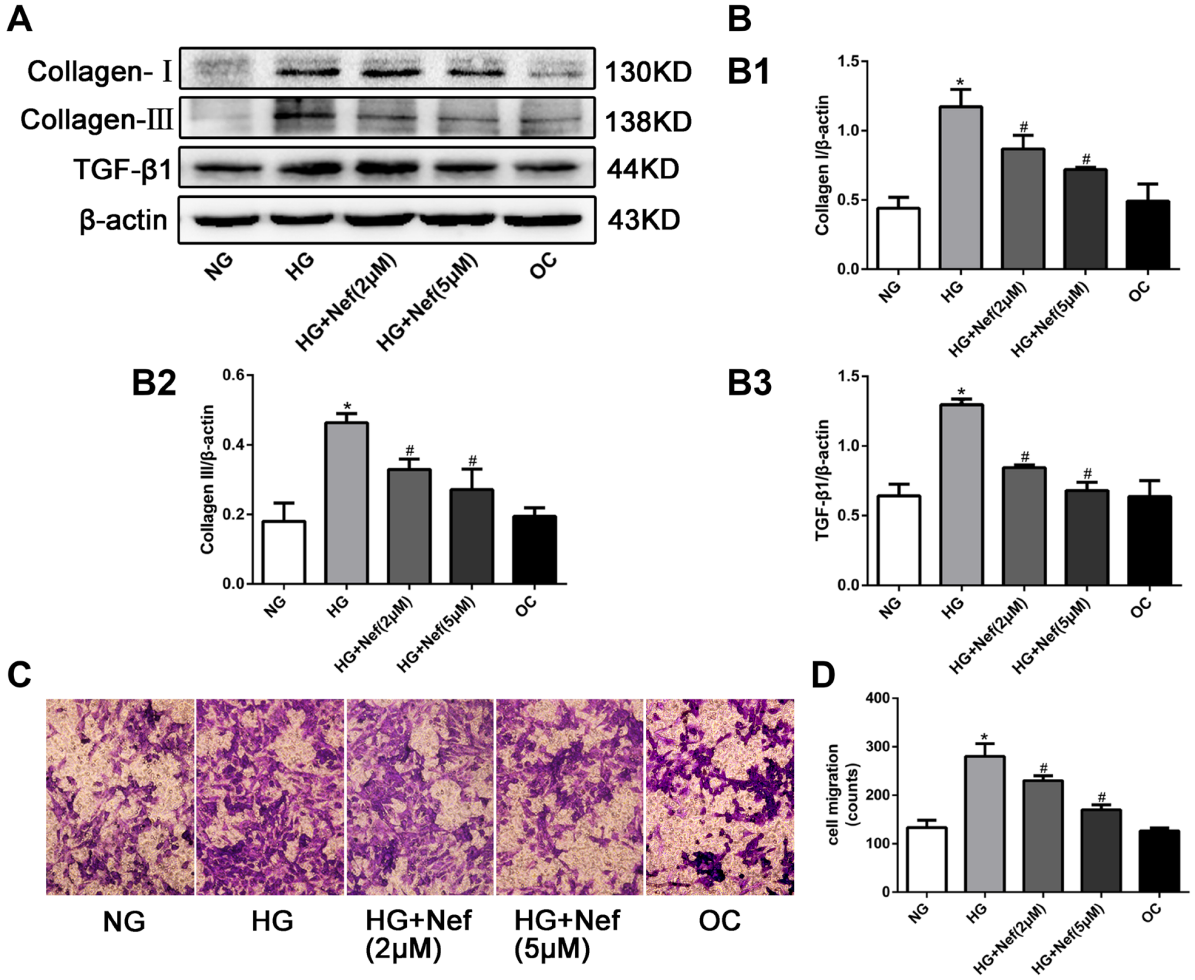
Neferine reduced the collagen deposition, down-regulated the protein expression of transforming growth factor β1 (TGF-β1), and inhibited the migration of CFs. (**A**) Western blot analysis of collagen I and III and TGF-β1 protein levels. (**B**) Quantitative analysis of the protein expression of collagen I and III and TGF-β1. (**C**) Transwell migration assay showed that neferine attenuated HG induced CFs migration. CFs were cultured in HG medium with neferine in 8-μm-pore-sized Transwell chamber for 10 h. CFs on the external surface of Transwell chamber were dyed with crystal violet and photographed under a microscope. (**D**) Quantification analysis of migration CF numbers in per filed of Transwell. NG: 5.6 mM glucose, HG: 30 mM glucose, HG+Nef (2 μM): 30 mM glucose + 2 μM neferine, HG+Nef (5 μM): 30 mM glucose + 5 μM neferine, OC: 5.6 mM glucose + 27.5 mM mannose. Data were means ± SD of three independent experiments. ^*^ *P* < 0.05 compared with the NG group; ^#^ *P* < 0.05 compared with the HG group.

