# Supplementary material for: Neferine inhibits proliferation and collagen synthesis induced by high glucose in cardiac fibroblasts and reduces cardiac fibrosis in diabetic mice
Source: Oncotarget. 2016 Aug 11;7(38):61703–15. doi: 10.18632/oncotarget.11225 (PMC5308684; doi:10.18632/oncotarget.11225)
Supplement: Supplementary file 1 [file oncotarget-07-61703-s001.pdf]

Neferine inhibits proliferation and collagen synthesis induced by high glucose in cardiac fibroblasts and reduces cardiac fibrosis in diabetic mice

SUPPLEMENTARY FIGURE

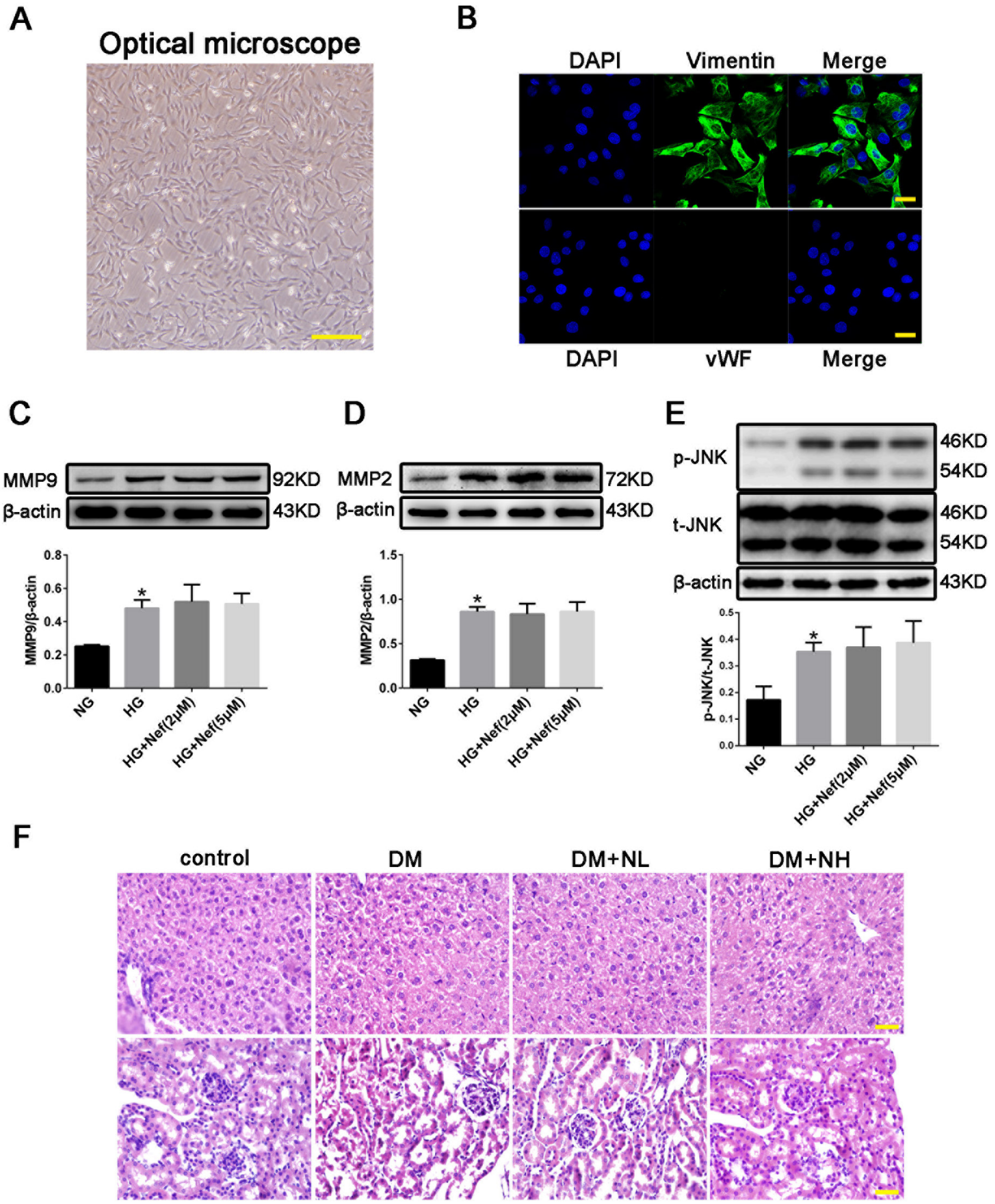

Supplementary Figure S1: A. CFs were observed under a microscope. Scale bar: 200 μm. **B.** Immunofluorescence staining with antibodies to Vimentin or Vwf. Scale bar: 25 μm. Representative western blot analysis of MMP9 and MMP2 (**C and D**) expression in CFs. **E.** Western blot analysis of the p-JNK/t-JNK protein expression. NG: 5.6 mM glucose, HG: 30 mM glucose, HG+Nef (2μM): 30 mM glucose + 2 μM neferine, HG+Nef (5μM): 30 mM glucose + 5 μM neferine. **F.** Representative hematoxylin-eosin (HE) staining of liver and kidney. Scale bar: 50 μm. Control: normal mice; DM: diabetic mellitus; DM+NL: DM mice with neferine administered at a dose of 60 mg/kg/day by gavage. DM+NH: DM mice with neferine administered at a dose of 120 mg/kg/day by gavage. Data were mean ± SD of three independent experiments. *P<0.05 compared with the NG group.
